# Supplementary material for: Combinatorial Effects of Cisplatin and PARP Inhibitor Olaparib on Survival, Intestinal Integrity, and Microbiome Modulation in Murine Model
Source: Int J Mol Sci. 2025 Jan 30;26(3):1191. doi: 10.3390/ijms26031191 (PMC11818058; doi:10.3390/ijms26031191)
Supplement: Supplementary file 1 [file ijms-26-01191-s001.zip › ijms-3393542-supplementary.pdf]

		Control	Olaparib	Cisplatin	Combination
jejunum	1M	397.8±105.7*	290.5 ± 61.1	348.1±67.8*	377.0 ± 83.2*
	3M	424 .4± 76.0	311.8 ± 74.2	397.7 ± 92.6	372.7 ± 69.1
	6M	413.6 ± 60.4	360.7 ± 86.0	375.7 ± 61.9	341.2 ± 62.5
ileum	1M	180.1±24.6*	162.1±21.7*	167.5 ± 18.1	181.9 ± 24.8
	3M	201.0 ± 37.0	139.1 ± 17.4	189.9 ± 42.0	165.5 ± 25.2
	6M	205.1 ± 72.4	137.6 ± 23.9	158.4 ± 21.9	161.3 ± 26.7

Table S1. Length(μm) of jejunal and ileal villi of four groups. Data are presented as *mean* ± *SEM*. Significance thresholds: *: $p < 0.05$.

		Control	Olaparib	Cisplatin	Combination
jejunum	1M	99.7±14.7	95.8±18.3	106.1±19.6	106.9±17.2
	3M	96.8±17.1	106.2±11.5	93.3±17.9	96.7±17.9
	6M	94.9±14.8	99.0±16.2	84.5±9.4	87.8±11.7
ileum	1M	69.5±5.4	82.8±22.0	66.9±15.6	79.7±13.3
	3M	94.7±32.1	75.6±9.1	58.7±9.9*	74.0±14.6
	6M	85.4±7.9	84.6±11.2	66.2±17.6	76.4±10.7

Table S2. Width(μm) of jejunal and ileal villi of four groups. Data are presented as *mean* ± *SEM*. Significance thresholds: *: $p < 0.05$.

		Control	Olaparib	Cisplatin	Combination
jejunum	1M	39922.3±10647.0	41858.7±8400.0	32797.7±8247.3	38631.3±7110.9
	3M	39570.7±8429.70	38001.8±11334.2	29818.5±6573.0	33021.5±5574.6
	6M	32394.5±4947.6	32901.0±4947.5	22847.3±7796.2	30024.8±6877.5
ileum	1M	10690.8±896.3	10973.7±3300.1	10331.8±1976.9	13799.2±3920.4
	3M	20026.0±12207.0	10890.5±2757.4	8973.2±2923.1*	9573.3±1091.9
	6M	13824.5±2679.6	13614.8±2480.7	8272.0±1032.4**	11239.0±2119.6

Table 3. Area(μm^2) of jejunal and ileal villi of four groups. Data are presented as *mean* ± *SEM*. Significance thresholds: *: $p < 0.05$, **: $p < 0.01$.

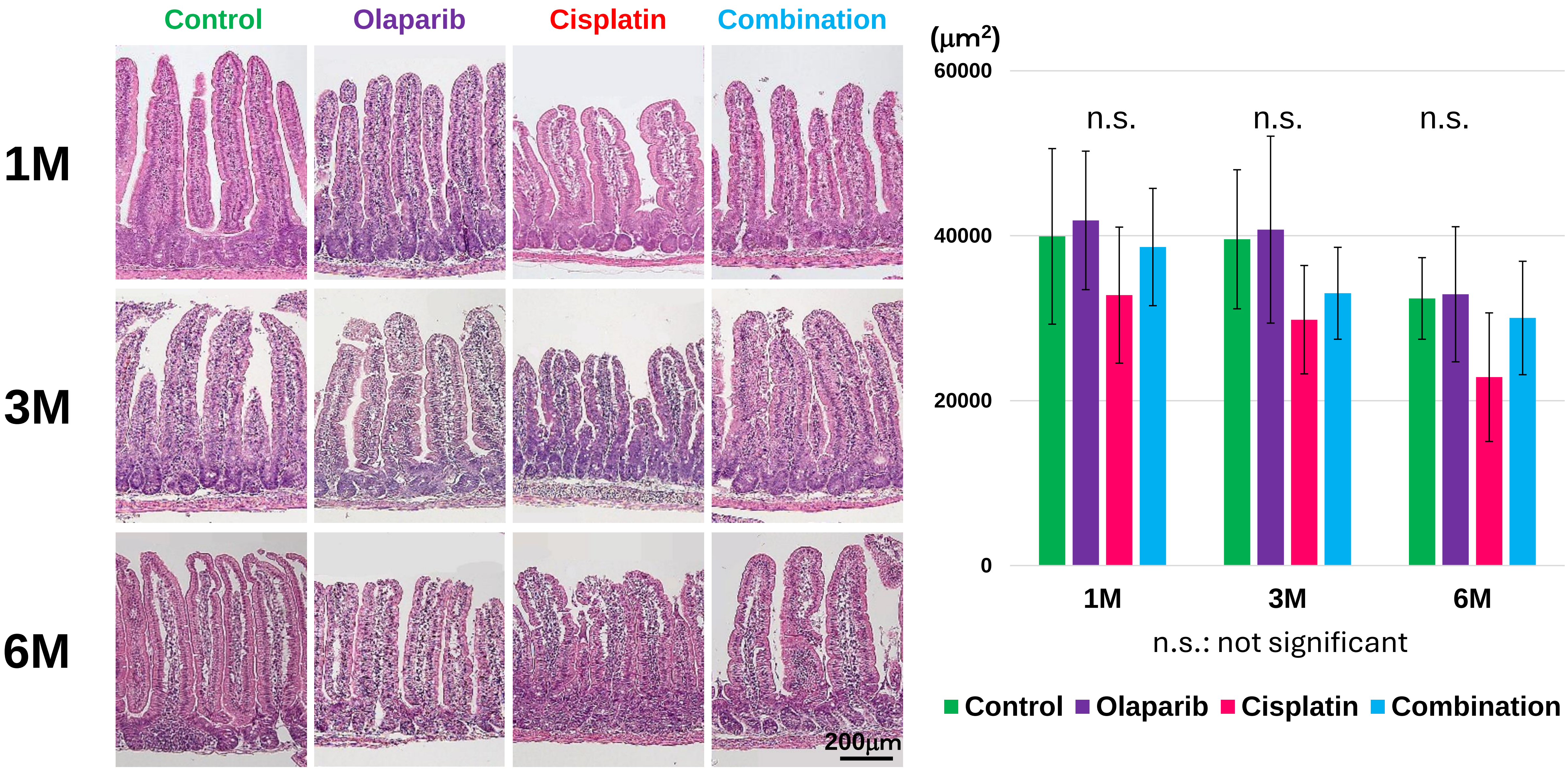

Figure S1: Histopathological changes of jejunum. (a) Representative hematoxylin and eosin (H&E) stained images of jejunal villi from each group at 1, 3, and 6 months after drug administration. Scale bar: 200 µm. (b) The villi area in the jejunum was quantified by measuring ten randomly selected regions from each section. Data are presented as mean ± SEM.

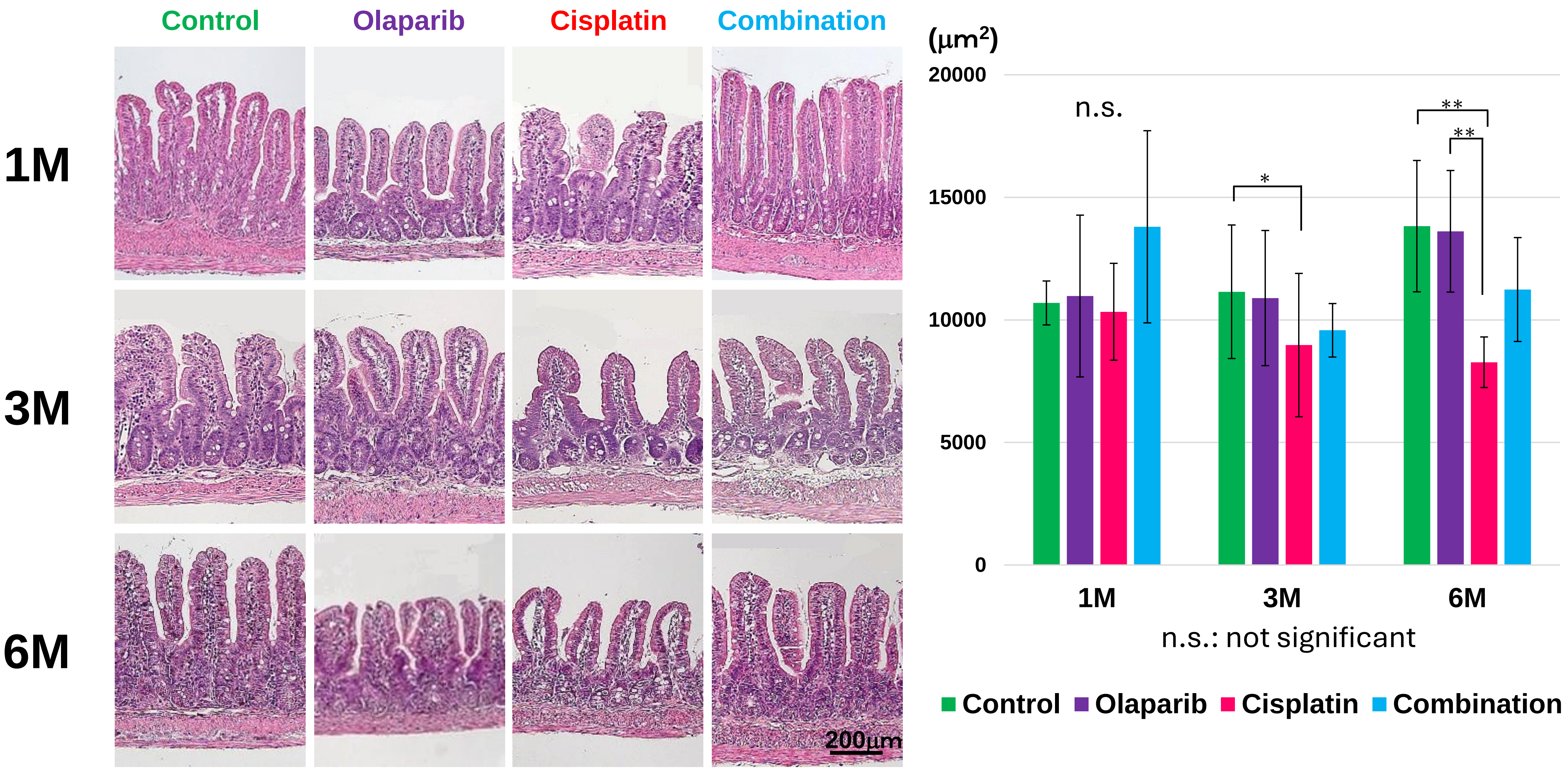

Figure S2: Histopathological changes of ileum. (a) Representative H&E-stained images of ileal villi from each group at 1, 3, and 6 months after drug administration. Scale bar: 200 μm. (b) The villi area in the ileum was quantified by measuring 10 randomly selected regions from each section. Data are expressed as mean ± SEM. *: $p < 0.05$, **: $p < 0.01$.

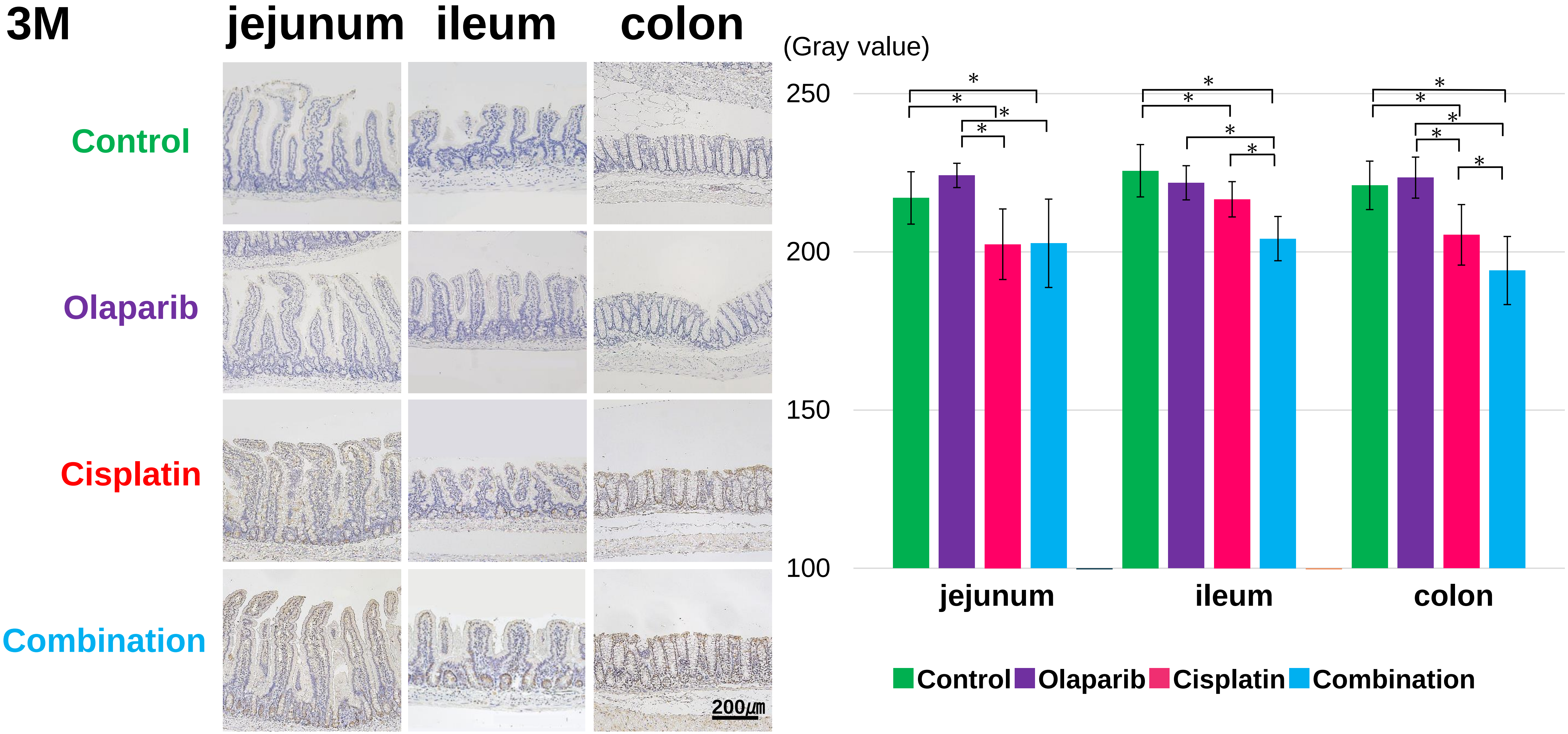

Figure S3: PARP-1 IHC at 3 month after drug administration. High-resolution IHC images and corresponding quantification bar graphs show strong PARP-1 positivity in the Cisplatin and combination groups compared to the control and Olaparib groups. Scale bar: 200 μm, Data are presented as mean ± SEM. * : $p < 0.05$.

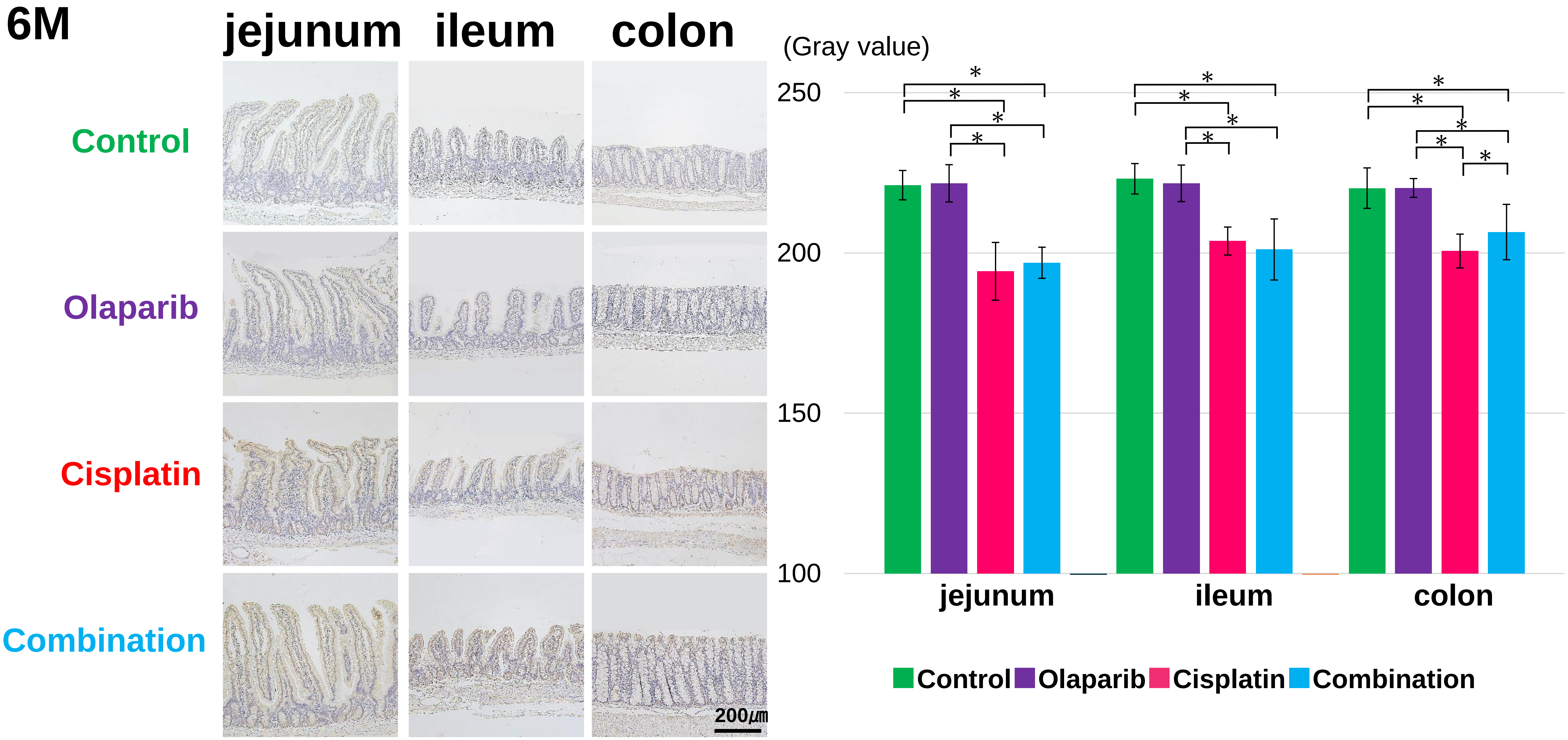

Figure S4: PARP-1 IHC at 6 month after drug administration. High-resolution IHC images and corresponding quantification bar graphs show strong PARP-1 positivity in the Cisplatin and combination groups compared to the control and Olaparib groups. Scale bar: 200 μm, Data are presented as mean ± SEM. * : $p < 0.05$.

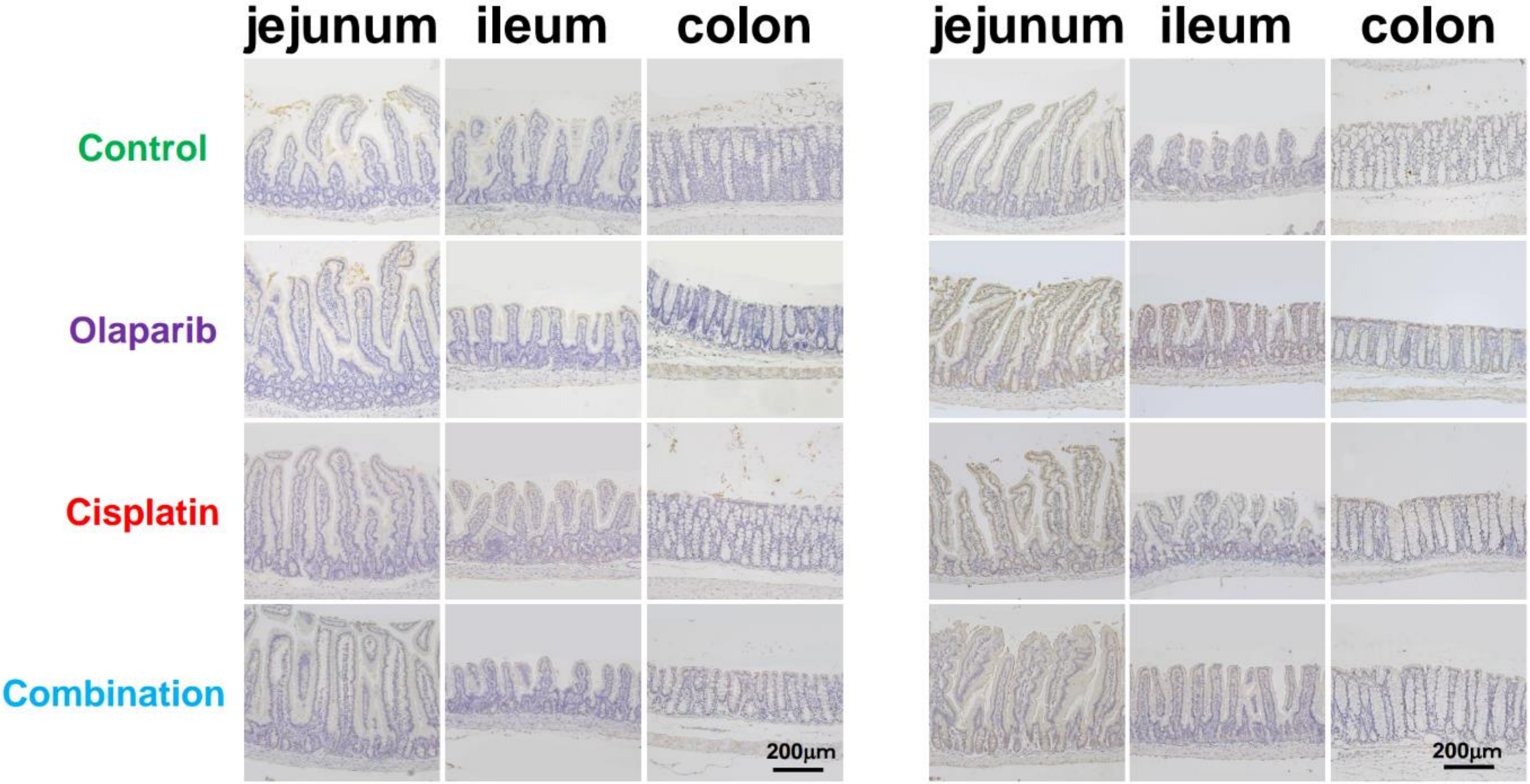

Figure S5: High resolution of IHC of EGF at (a) 3 and (b) 6 months after completion of drug administration, showing no clear pattern across the groups or drug administration.

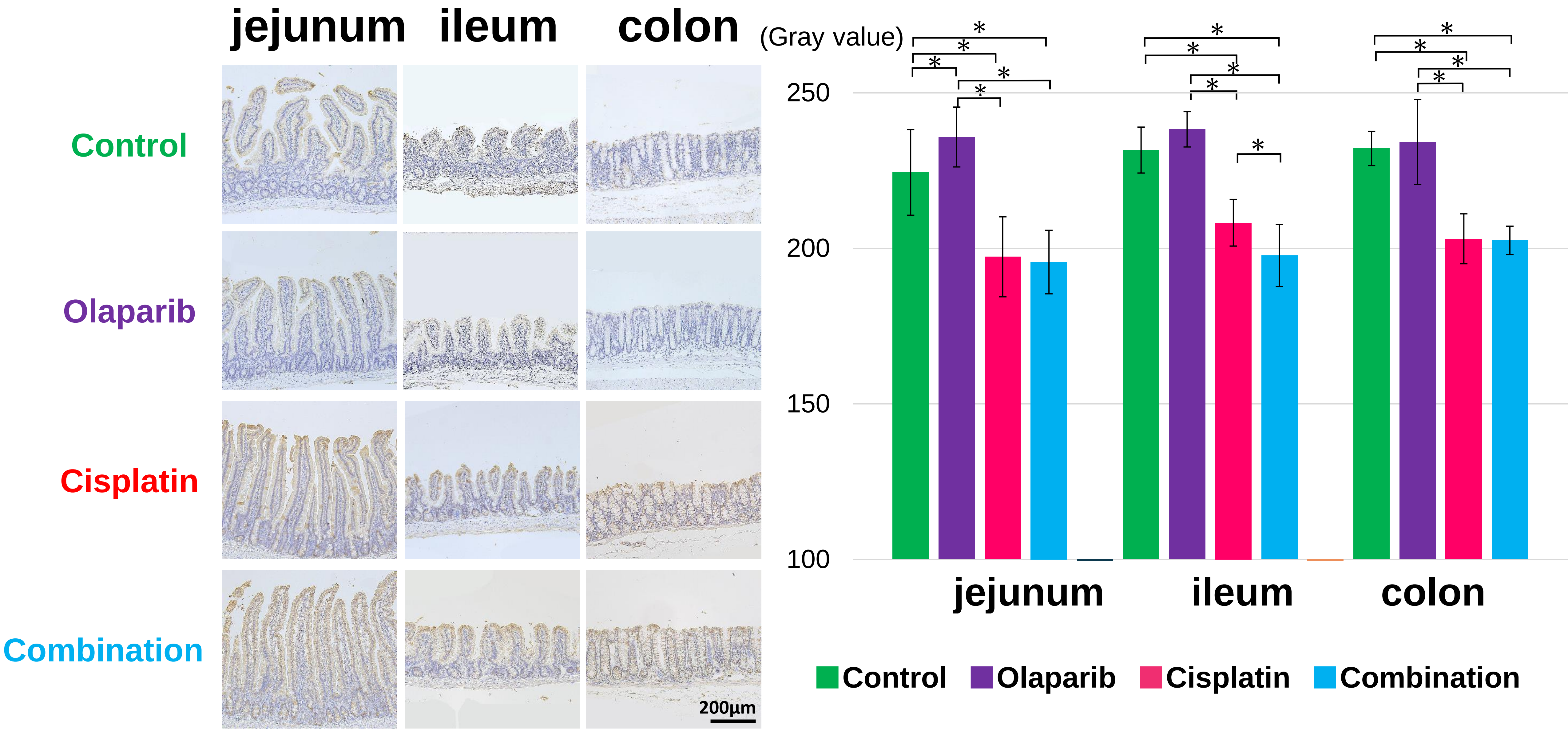

Figure S6: High resolution of IHC of E-cadherin at 3 months after completion of drug administration. (a) representative photos from each group and (b) corresponding semi-quantitative bar graphs. Scale bar: 200 µm, Data are presented as mean ± SEM. * : $p < 0.05$.

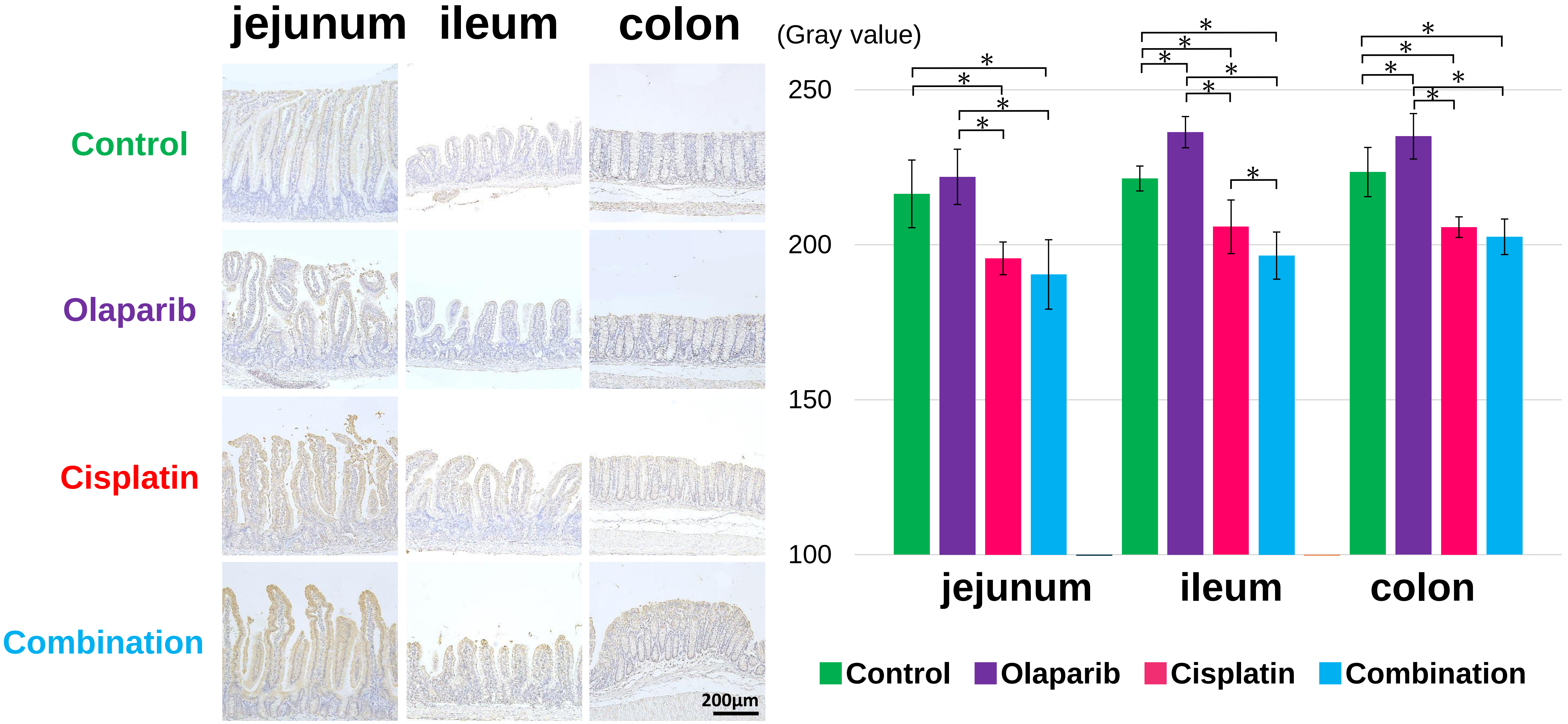

Figure S7: High resolution of IHC of E-cadherin at 6 months after completion of drug administration.
(a) representative photos from each group and (b) corresponding semi-quantitative bar graphs.
Scale bar: 200 µm, Data are presented as mean ± SEM. * : $p < 0.05$.

	level	Control	Olaparib	Cisplatin	Combination
1M	phylum	2	2	2	2
	class	3	3	3	3
	order	3	4	4	4
	family	5	5	6	6
	genus	5	5	4	5
	species	6	6	4	6
3M	phylum	2	2	2	2
	class	3	3	3	3
	order	4	4	3	3
	family	5	5	5	5
	genus	6	5	6	4
	species	7	8	6	6
6M	phylum	2	2	2	2
	class	2	2	2	2
	order	3	3	3	3
	family	4	4	4	4
	genus	7	5	6	6
	species	7	4	5	6

Table S4: Relative abundance of gut microbiota more than 5%.

	level	Control	Olaparib	Cisplatin	Combination
1M	phylum	7	7	6	7
	class	8	8	7	8
	order	17	17	16	16
	family	26	25	24	24
	genus	48	49	34	43
	species	57	62	47	55
3M	phylum	8	7	7	7
	class	9	8	8	8
	order	16	16	16	14
	family	25	25	24	22
	genus	54	46	44	45
	species	64	60	61	53
6M	phylum	7	7	7	7
	class	7	7	7	8
	order	14	15	14	15
	family	23	24	24	24
	genus	53	54	53	55
	species	51	53	52	54

Table S5: Relative abundance of gut microbiota more than 0.1%.

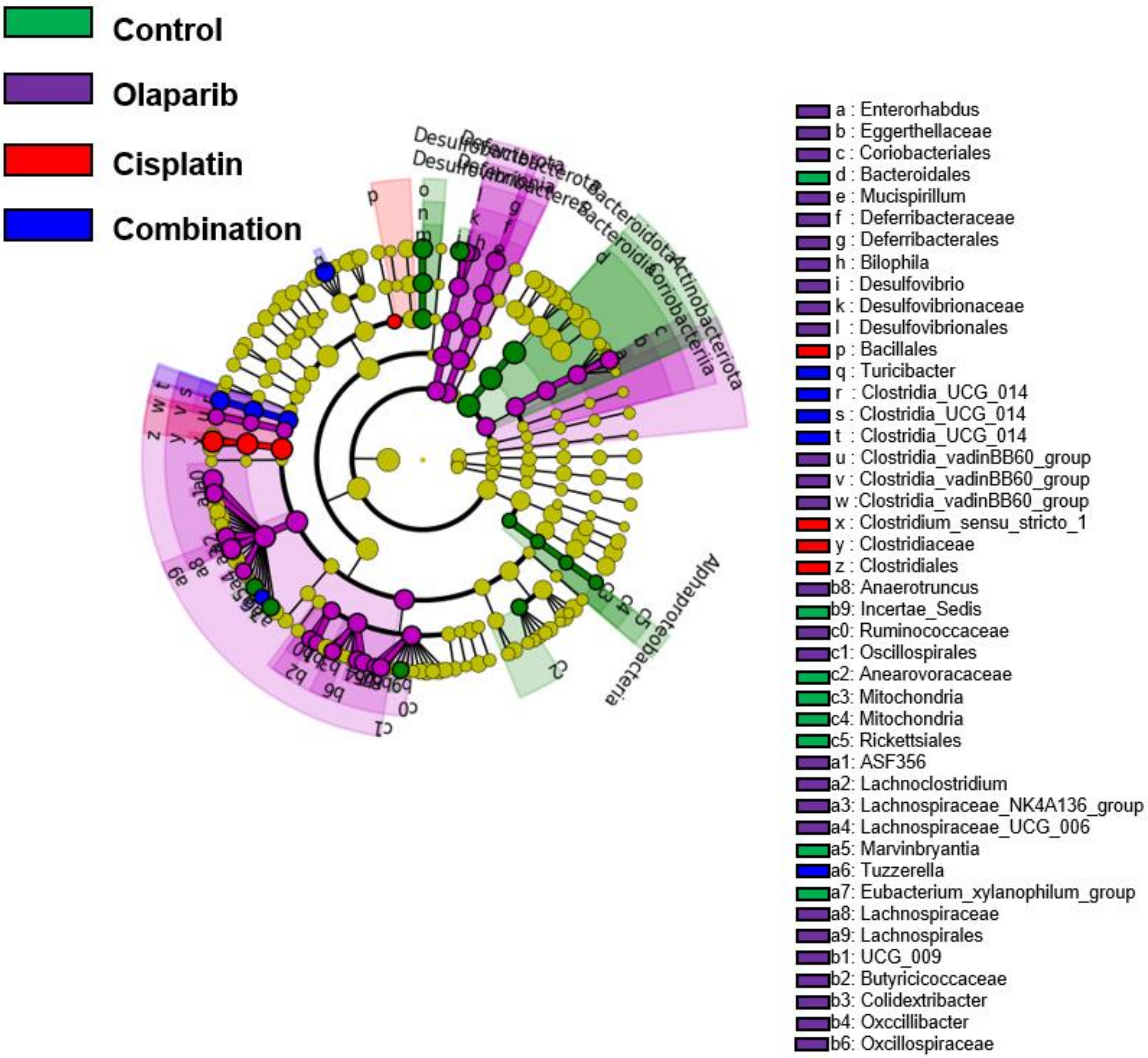

Figure S8: High-resolution cladogram plotted from LEfSe analysis at 1 month after drug administration.

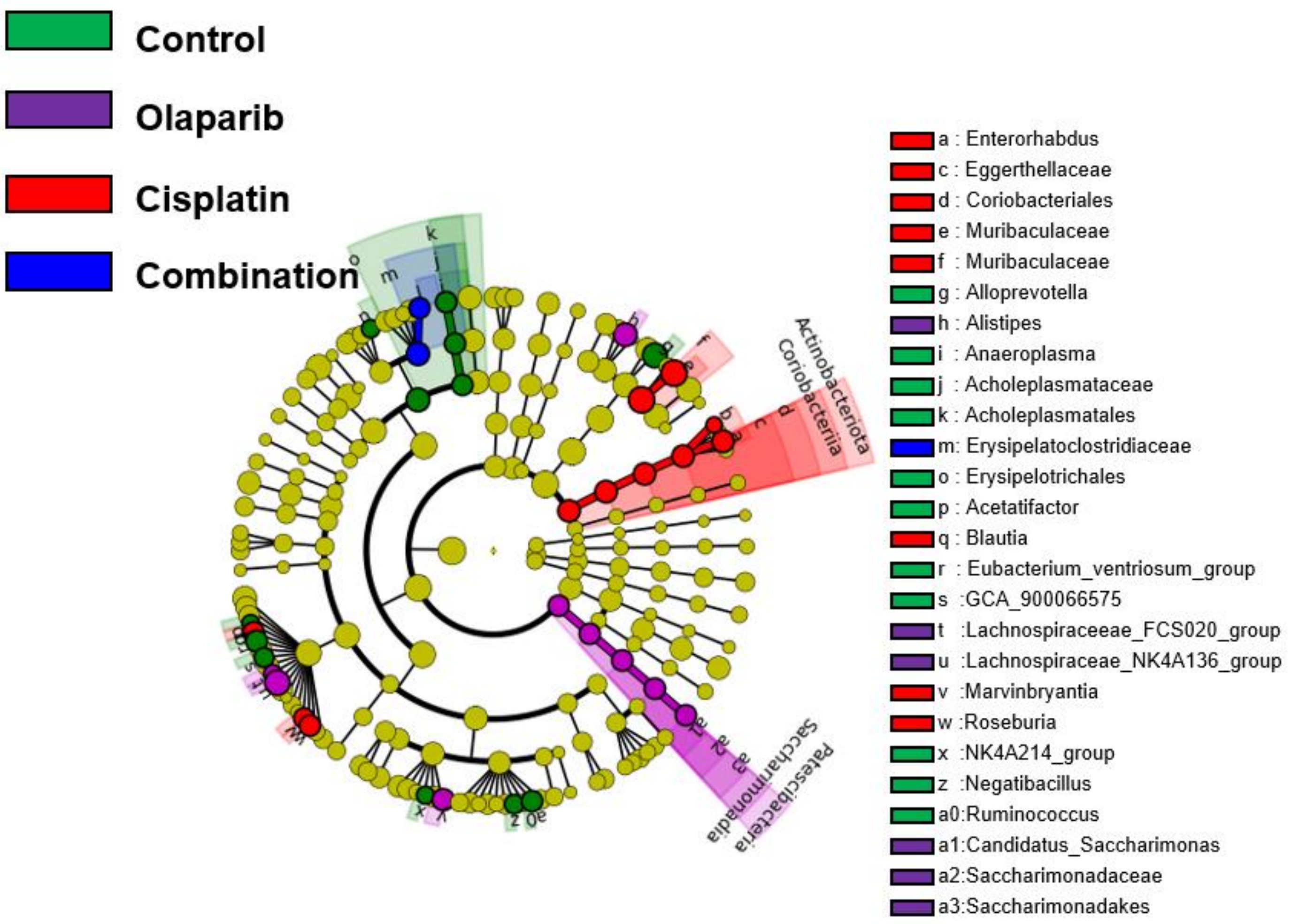

Figure S9: High-resolution cladogram plotted from LEfSe analysis at 3 months after drug administration.

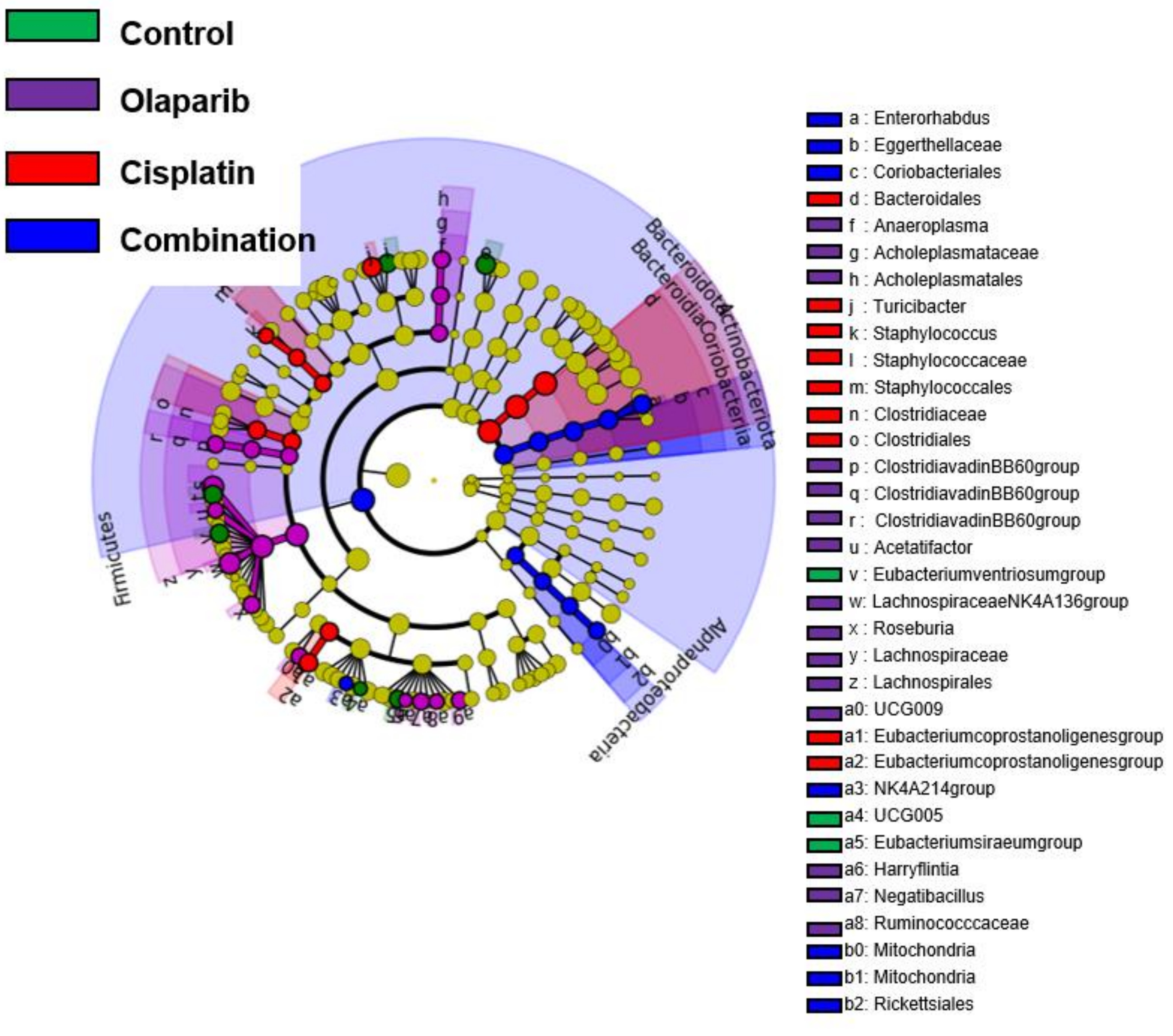

Figure S10: High-resolution cladogram plotted from LEfSe analysis at 6 months after drug administration.

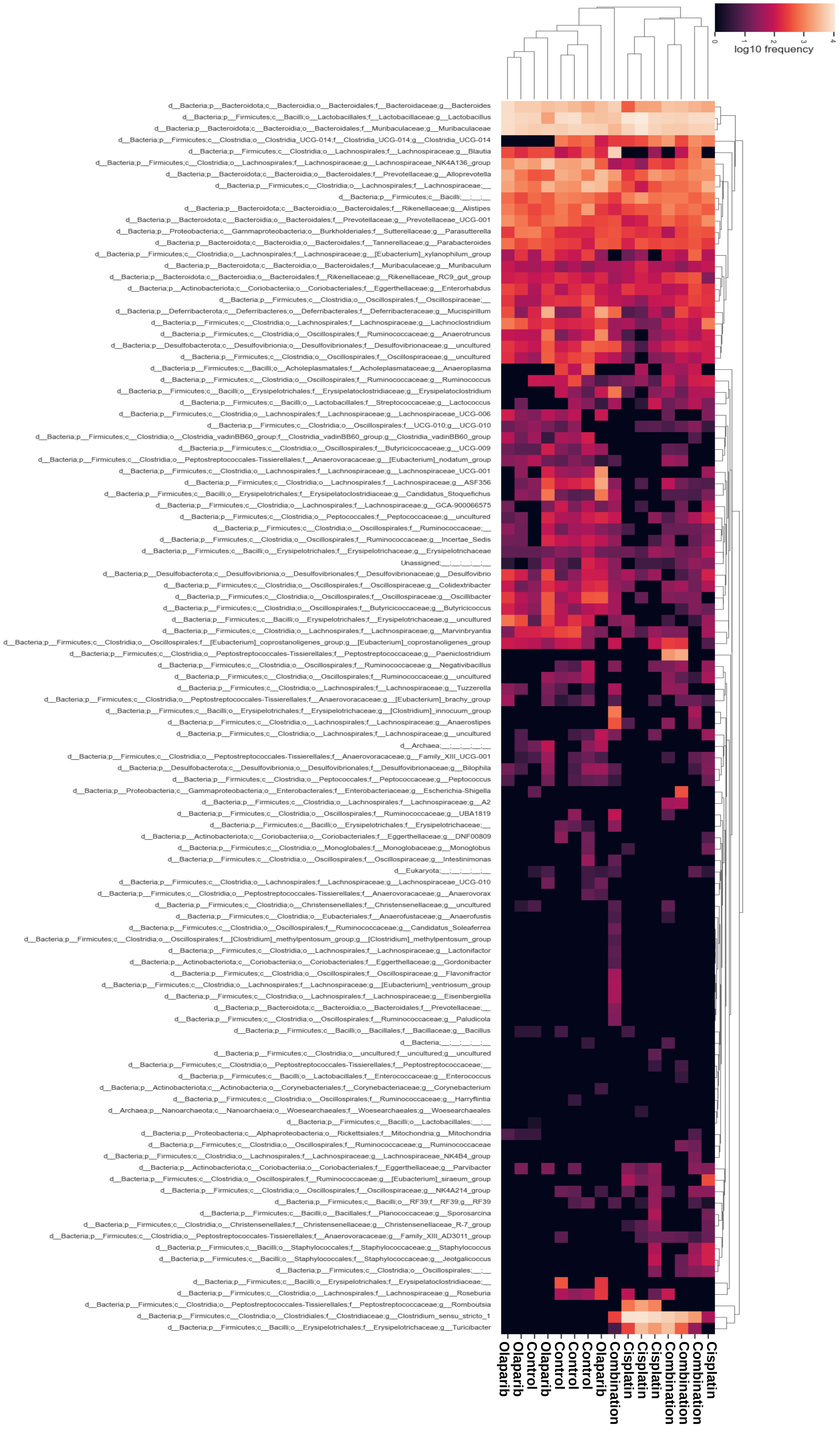

Figure S10: Whole heatmap analysis at 1 months after drug administration

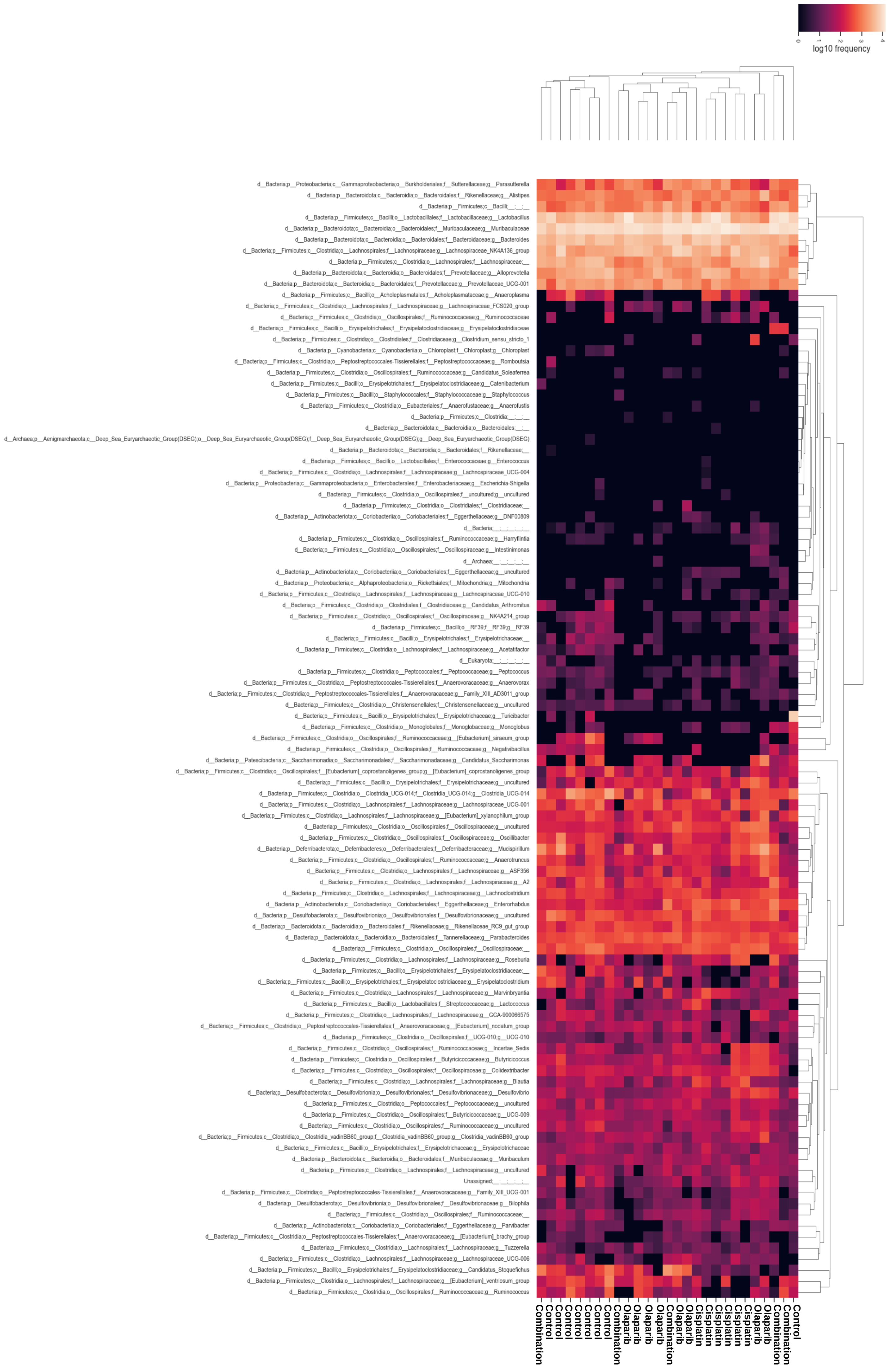

Figure S11: Whole heatmap analysis at 3 months after drug administration

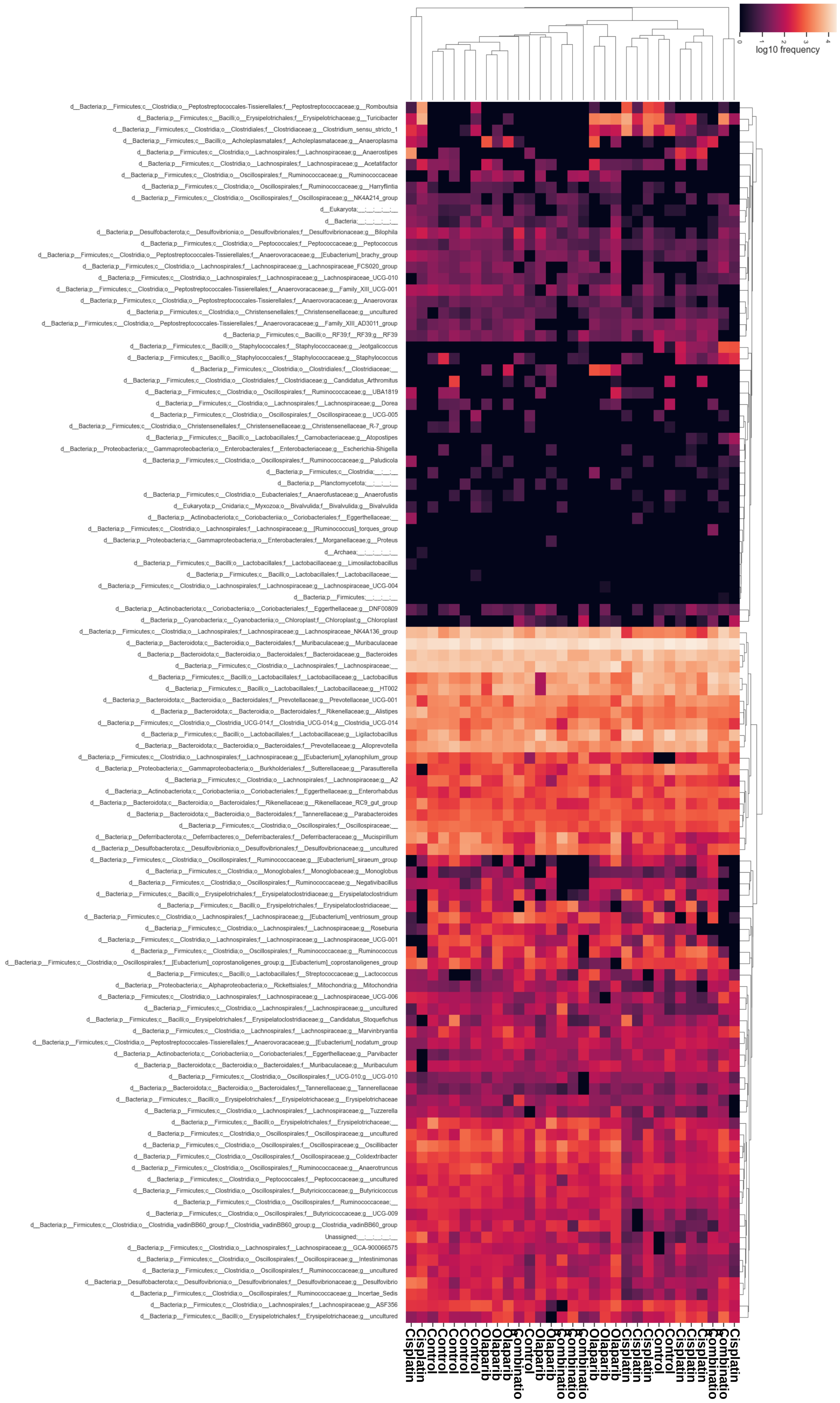

Figure S12: Whole heatmap analysis at 1 months after drug administration
